# Differential Roles of Environmental Enrichment in Alzheimer’s Type of Neurodegeneration and Physiological Aging

**DOI:** 10.3389/fnagi.2017.00245

**Published:** 2017-07-26

**Authors:** Vladimir V. Salmin, Yulia K. Komleva, Natalia V. Kuvacheva, Andrey V. Morgun, Elena D. Khilazheva, Olga L. Lopatina, Elena A. Pozhilenkova, Konstantin A. Shapovalov, Yulia A. Uspenskaya, Alla B. Salmina

**Affiliations:** ^1^Department of Medical and Biological Physics, Krasnoyarsk State Medical University named after Prof. V.F. Voino-Yasenetsky Krasnoyarsk, Russia; ^2^Department of Biochemistry, Medical, Pharmaceutical and Toxicological Chemistry, Krasnoyarsk State Medical University named after Prof. V.F. Voino-Yasenetsky Krasnoyarsk, Russia; ^3^Research Institute of Molecular Medicine and Pathobiochemistry, Krasnoyarsk State Medical University named after Prof. V.F. Voino-Yasenetsky Krasnoyarsk, Russia; ^4^Department of Pediatrics, Krasnoyarsk State Medical University named after Prof. V.F. Voino-Yasenetsky Krasnoyarsk, Russia

**Keywords:** environmental enrichment, neurogenesis, neurosphere, Alzheimer’s disease, aging, time-lag model

## Abstract

Impairment of hippocampal adult neurogenesis in aging or degenerating brain is a well-known phenomenon caused by the shortage of brain stem cell pool, alterations in the local microenvironment within the neurogenic niches, or deregulation of stem cell development. Environmental enrichment (EE) has been proposed as a potent tool to restore brain functions, to prevent aging-associated neurodegeneration, and to cure neuronal deficits seen in neurodevelopmental and neurodegenerative disorders. Here, we report our data on the effects of environmental enrichment on hippocampal neurogenesis *in vivo* and neurosphere-forming capacity of hippocampal stem/progenitor cells *in vitro.* Two models – Alzheimer’s type of neurodegeneration and physiological brain aging – were chosen for the comparative analysis of EE effects. We found that environmental enrichment greatly affects the expression of markers specific for stem cells, progenitor cells and differentiated neurons (Pax6, Ngn2, NeuroD1, NeuN) in the hippocampus of young adult rats or rats with Alzheimer’s disease (AD) model but less efficiently in aged animals. Application of time-lag mathematical model for the analysis of impedance traces obtained in real-time monitoring of cell proliferation *in vitro* revealed that EE could restore neurosphere-forming capacity of hippocampal stem/progenitor cells more efficiently in young adult animals (fourfold greater in the control group comparing to the AD model group) but not in the aged rats (no positive effect of environmental enrichment at all). In accordance with the results obtained *in vivo*, EE was almost ineffective in the recovery of hippocampal neurogenic reserve *in vitro* in aged, but not in amyloid-treated or young adult, rats. Therefore, EE-based neuroprotective strategies effective in Aβ-affected brain could not be directly extrapolated to aged brain.

## Introduction

Environmental enrichment (EE) is considered as an environment with numerous sensorimotor, cognitive, and social stimulations able to affect brain plasticity, to restore brain functional reserves, and to facilitate establishment of novel connections actual for preventive or rehabilitation strategies. Modeling EE is currently widely used in experiments aimed to study synaptogenesis, neurogenesis, and brain connectivity (Komleva Iu et al., 2013). EE stimulates neurogenesis and synapse turnover, integration of newly-formed cells into neuronal ensembles, modifies epigenetic mechanisms controlling resistance to oxidative stress, modulates production of neurotransmitters and molecules with neurotrophic activity, prevents apoptosis, suppresses neuroinflammation, affects neuron-glia interactions, thereby enhancing cognition, learning, and social communications in animals or humans with or without brain pathology (Herring et al., 2011; Cotel et al., 2012; Komleva Iu et al., 2013; Leger et al., 2015; Grinan-Ferre et al., 2016; Stuart et al., 2017). These effects well-correspond to those observed in people practicing so-called cognitive training because of their professional duties or personal habits and demonstrating good preservation of cognitive functions even at the eldest period of life (Mora, 2013). However, action of EE depends on experimental conditions, duration of exposure, severity of brain injury, age, gender etc., thereby, the observed effects of EE on neuroplasticity might be different and even unexpected (Ming and Song, 2011; Valero-Aracama et al., 2015).

Growing number of experimental and clinical findings suggest that EE might serve as an effective strategy to restore the functional capacity of damaged brain, i.e., in Alzheimer’s disease (AD), in neurodevelopmental disorders, after stroke, etc. (Janssen et al., 2014; Polito et al., 2014; Cioni et al., 2016; Rosbergen et al., 2016). Particularly, prevention or treatment of Alzheimer-type neurodegeneration is one of the most challenging questions in the modern neuroscience which is directly linked to the controlled modulation of hippocampal plasticity (Balietti et al., 2012) In such context, EE provides a lot of premises for its effective application: prevention of hippocampal astroglial dysfunction in the AD transgenic mice (Rodriguez et al., 2013), up-regulation of brain-derived growth factor expression in the hippocampus of senescence-accelerated prone mice (Yuan et al., 2012), prevention of amyloid β (Aβ) deposition and memory impairment in AD model mice (Maesako et al., 2012), modulation of hippocampal synaptic proteins expression (Barak et al., 2013).

In contrast, EE action in aging brain is not deciphered in detail, even aging itself is the certain risk for AD development (Avila et al., 2010). Moreover, precise molecular mechanisms of EE effects on neuroplasticity in aged or AD brain remain to be unclear or even contradictory (Herring et al., 2011; Cotel et al., 2012; Bezzina et al., 2015; Huttenrauch et al., 2016). Impairment of hippocampal adult neurogenesis in aging is a well-known phenomenon caused by the shortage of brain stem cell pool, alterations in the local microenvironment within the neurogenic niches, or deregulation of stem cell development (Ali et al., 2015; Moraga et al., 2015). It is well-known that there is an obvious contrast between Alzheimer-type neurodegeneration and physiological aging, however, both of them are associated with progressive cognitive deficits and impairment of brain plasticity. Healthy aging brain is characterized by moderate decline in neurogenesis affecting the structure and function of the entorhinal-hippocampal circuit which is in the focus of neurodegeneration seen in AD due to the toxic action of Aβ, oxidative stress, excitotoxicity, and neuroinflammation (Hollands et al., 2016). In physiologically aging brain, no significant changes in the number of neural stem cells (NSCs) residing within the hippocampal neurogenic niche have been detected (Hattiangady and Shetty, 2008). In contrast, gradual decrease of NSCs and neural progenitor cells (NPCs) number has been reported in the experimental APP/PS1/nestin-GFP triple transgenic mouse model of AD (Zeng et al., 2016). Thus, the earliest stages of adult neurogenesis (stem cell maintenance, self-renewal, and proliferation) are significantly affected in AD neurodegeneration but not in healthy aging. Since enhancing neurogenesis was proposed as a therapeutic approach in neurodegeneration (Hollands et al., 2016), one may assume that EE could produce different effects on aging-associated and AD-compromised hippocampal neurogenesis. Despite several experimental and clinical observations on EE action in elderly subjects with or without AD, little information is available on the comparative efficacy of EE in AD and healthy aging.

So, whether EE-induced changes are equally beneficial in aging brain and in AD? The subject has received increased attention with the deciphering the biological mechanisms of late-life brain alterations: reduced neurogenesis and synapse turnover (Mostany et al., 2013), suppressed release of neuropeptides and neurotrophic factors (Forlenza et al., 2015), appearance of white matter lesions and pathological blood–brain barrier permeability (Marin-Padilla and Knopman, 2011; Firbank et al., 2012). Several studies suggest that EE affects epigenetic mechanisms controlling neuroplasticity, promotes remyelination, or reduces glia-supported neuroinflammation in the neurogenic niches in the aged brain (Williamson et al., 2012; Yang et al., 2012; Neidl et al., 2016). However, alterations in neurogenesis and the capacity of so-called “neurogenic reserve” may have different patterns and degree of progression in normal aging and in AD (Esiri and Chance, 2012).

Assessment of neurogenesis represents an appropriate system to analyze the activity of factors with presumptive neuroprotective properties. *In vivo*, neurogenesis can be studied with the approach based on the detection of markers expression in cells at different stages of development within the neurogenic niches or along their migratory paths in the brain (Roybon et al., 2009). Apart from this, some neurogenesis-associated events are easily reproduced *in vitro* due to ability of neurogenic non-differentiated cells to produce non-adherent spherical clusters of cells known as neurospheres (NS). NS-forming capacity is a parameter which is widely used for the assessment of neurogenic potential of neural stem/progenitor cells as well as the action of regulatory molecules, neurotoxic substances, or drug candidates (Pacey et al., 2006). Positive effects of EE on neurogenesis *in vivo* have been shown (Monteiro et al., 2014; Clemenson et al., 2015), however, there are very limited data on NS development *in vitro* in AD or aging (Heo et al., 2007; Diaz-Moreno et al., 2013).

Recently, we have demonstrated EE-mediated enhancement of neurosphere-forming capacity of NSCs obtained from the brains of AD rat model (Kuvacheva et al., 2015). It should be mentioned that analysis of data obtained with NS culture systems is hampered by the relative shortage of tools for adequate interpretation within the context of cell cycle- and proliferation-related events. Modeling the cell growth kinetics has been in the focus of researchers for a long time (Baker et al., 1998), but mathematic analysis of kinetic features of stem cells behavior in several subsequent NS generations *in vitro* was undertaken in few studies only (Ma et al., 2007). Development of correct mathematical models of NS establishment in the short-term experimental conditions (when reduction of cell population due to cell death might be contingently neglected) would provide new opportunities for rapid and effective analysis of endogenous and exogenous stimuli targeting neurogenesis. Moreover, current achievements in the application of NS cultures for AD modeling *in vitro* (Choi et al., 2013; Ghate et al., 2014) or novel attempts to restore brain functions with NSCs transplantation therapy (Waldau and Shetty, 2008) determine the needs in the appropriate mathematical modeling of NS-generation *in vitro* to clarify mechanisms underlying neurogenesis modulation in a given microenvironment.

Thus, we may entertain the hypothesis that: (i) effects of EE on neurogenesis are different in healthy brain aging and in brain affected by Alzheimer’s type of neurodegeneration; (ii) combination of *in vivo* and *in vitro* approaches to the assessment of neurogenesis with the mathematical modeling of neurogenic reserve could help us to identify the neurogenesis stage which is a main “target” for the action of EE in normal and damaged brain. Therefore, the goal of this study was to compare the effects of *in vivo* EE in the Alzheimer’s type of neurodegeneration and physiological brain aging with the special focus on hippocampal neurogenesis *in vivo* and NS-forming capacity of hippocampal stem/progenitor cells *in vitro*.

### Experimental Procedures

#### Modeling Environmental Enrichment *In Vivo*

Wistar male rats (*n* = 64) were maintained in standard cages with water and standard diet with free access to food and water. The following groups of animals were included in the experiment: (i) young adult rats, 7–9 months old (*n* = 40) kept under standard conditions (SC, *n* = 20) or in the environmental enrichment (EE, *n* = 20); (ii) elderly rats, 23–25 months old (*n* = 24) kept under SC or in the EE. In SC, rats were housing in the cages sized 50 cm × 30 cm × 18 cm (five animals in one cage). In EE, rats were housing for 60 days in cages sized 78 cm × 48 cm × 39 cm (10 animals in one cage) equipped with various devices to provide extensive physical and explanatory activity (tunnels, houses, hammocks, stairs, boxes, wheels) (Jankowsky et al., 2003).

#### Modeling Alzheimer-Type Neurodegeneration

In the group of young adult rats, 10 animals kept under SC and 10 animals kept in EE have been used for modeling AD with intracerebral bilateral stereotaxic-guided (Narishige Scientific Instrument Lab) injections of 5 μl (2 μg/μl) aggregated Aβ1-42 (Sigma-Aldrich) into CA1 zones of hippocampus at the day 60 of SC or EE housing according to the protocol described (Li et al., 2011). For this procedure, anesthesia with chloral hydrate (0.35 g/kg) has been applied, and the following stereotaxic coordinates have been chosen: A/P = 3.0 mm, M/L = ± 2.2 mm, D/V = 2.8 mm. Establishment of AD model (Aβ deposition and cognitive impairment) has been confirmed with Thioflavin S staining of brain tissue and neurobehavioral phenotyping of animals (data not shown).

#### Assessment of Hippocampal Neurogenesis

Expression of markers in NSCs, progenitor cells and differentiated neurons (Pax6, Neurogenin 2, NeuroD1, NeuN) was assessed in the hippocampus of animals in all experimental groups according to the standard protocol of immunohistochemistry with antigen unmasking (proteinase K) procedure. Briefly, 2 μm hippocampal slices were obtained after transcardial perfusion of anesthesized rats with 0.1 M PBS (pH 7.4) and 4% PFA followed by hippocampus fixation in 10% buffered PFA for 24 h. The following primary antibodies have been used: Pax-6 (Abcam, ab78545, mouse monoclonal), 1:100, Neurogenin2 (Abcam, ab26190, rabbit polyclonal), 1:50, NeuroD1 (Abcam, ab60704, mouse monoclonal), 1:100, NeuN (Millipore, ABN78, rabbit polyclonal), 1:500. Alexa-conjugated secondary antibodies were used in 1:500 dilution: Alexa Fluor 488 chicken anti-mouse (for Pax6 detection), Alexa Fluor 488 donkey anti-rabbit (for Neurogenin2 detection), NeuN (rabbit polyclonal) – Alexa Fluor 488 donkey anti-rabbit (for NeuroD1 detection), and Alexa Fluor 488 chicken anti-rabbit (for NeuN detection) on slices. Images were visualized with Olympus CX41 microscope and analyzed with Image J software. Corrected total cell fluorescence (CTCF) was calculated for all the images.

#### Isolation of Adult Hippocampal Cells with the NS-Forming Capacity

At the day 70 of housing (10 days after AD modeling in the corresponding group of animals), isolation of hippocampus was performed in anesthesized rats according to the standard protocol described (Kuvacheva et al., 2015). After brain tissue dissociation and isolation of hippocampal cells, counting of the cells for further adjusting the viable cell concentrations was done with the Scepter CellCounter (Millipore). Assessment of proliferation of obtained hippocampal cells *in vitro* has been performed by culturing them in NeuroCult^®^NS-A Proliferation Medium with bFGF and EGF (Stemcell). On the next day after cell plating, development of neurospheres as transparent free-floating cell aggregates with surface microspikes was assessed with phase-contrast microscopy. At the day 3 of NS culture, cells were carefully harvested from the flasks, centrifuged, and suspended in the fresh NeuroCult^®^NS-A Proliferation Medium.

#### Real-Time Analysis of Cell Proliferation *In Vitro*

Further real-time analysis of cell proliferation kinetics was done using xCElligence system (Roche) with the gold microelectrode-covered microtiter plates. This method allows electrical impedance monitoring in the real-time manner. For this, 1.2–2.5 × 10^4^ cells/ml were cultured in NeuroCult^®^NS-A Proliferation Medium (100 μl per well, four wells for each series) at +37°C, 5% CO_2_ with the measurements intervals of 5 min (from time 0 to 4.5 h) and 15 min (from 4.5 to 24 h).

### Statistical Analysis

Non-linear regression analysis was done with the Origin 8.5 software (OriginLab). We applied user-defined function models. All regression models indicate the confidence coefficient χ^2^ not worsen than 10^-4^, and determination coefficient *R*^2^ not less than 0.99. Statistical analysis was performed with the GraphPad Prism 6.0 software. Statistical significance was determined by one-way or two-way ANOVA with a Sidak’s test multiple comparisons test. The data are presented as mean and standard deviation.

## Results

**FIGURE 1:**
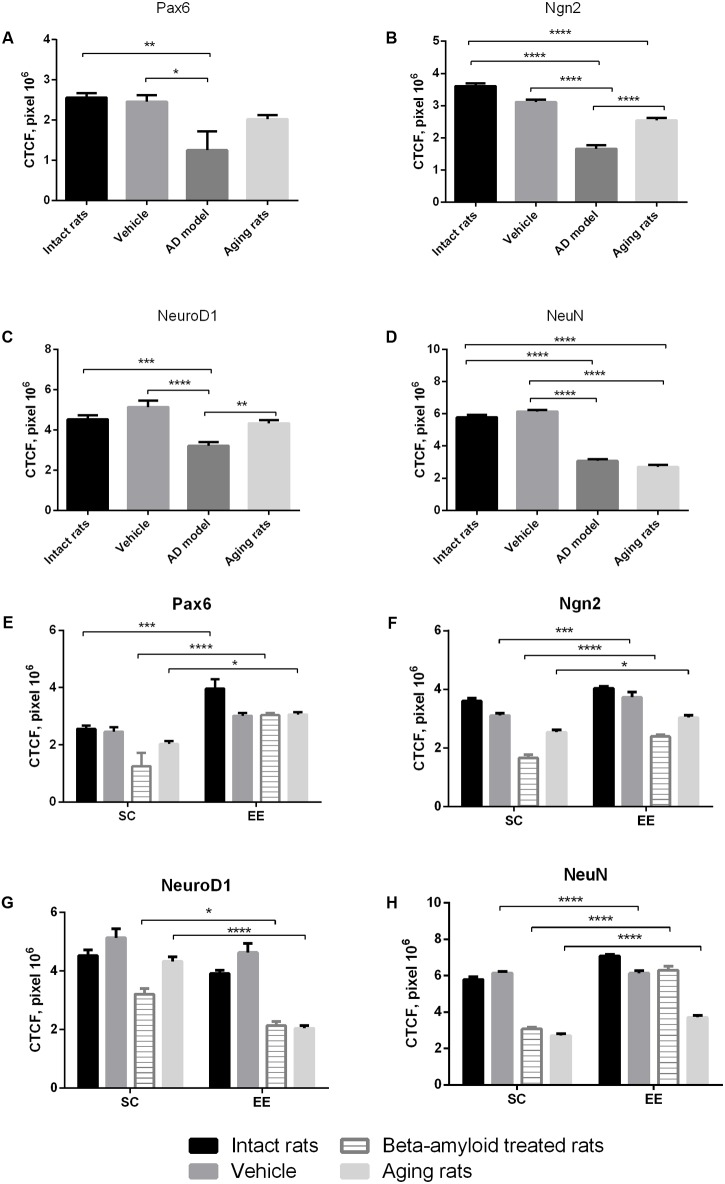
Neurogenesis in the subgranular and granular layers of the dentate gyrus in experimental groups (intact rats, vehicle – sham-operated rats, AD model – Aβ-treated rats, aging rats, SC – standard conditions, EE – environmental enrichment). **(A–D)** Measurement of the CTCF index of different neurogenesis markers between groups under standards conditions (*N* = 10/per group, one-way ANOVA; Turkey’s multiple comparisons test was run between groups). **(A)** Pax6 CTCF index (*p* < 0.01; one-way ANOVA). **(B)** Ngn2 CTCF index (*p* < 0.0001; one-way ANOVA). **(C)** NeuroD1 CTCF index (*p* < 0.0001; one-way ANOVA). **(D)** NeuN CTCF index (*p* < 0.0001; one-way ANOVA). Asterisks indicate statistical differences between groups. ^∗^*p* < 0.05; ^∗∗^*p* < 0.01; ^∗∗∗^*p* < 0.001; ^∗∗∗∗^*p* < 0.0001. **(E–H)** Measurement of the CTCF index of different neurogenesis markers between groups under standards conditions compared to environmental enrichment (*N* = 10/per group, statistical significance was determined by two-way ANOVA with a Sidak’s test multiple comparisons test). **(E)** Pax6 CTCF index [Interaction *p* < 0.05, *F*(3,72) = 2.573, two-way ANOVA]. **(F)** Ngn2 CTCF index [Interaction ns; environmental factor *p* < 0.0001, *F*(1,72) = 68.85, group factor *p* < 0.0001, *F*(3,72) = 130.7, two-way ANOVA]. **(G)** NeuroD1 CTCF index [Interaction *p* = 0.0001, *F*(3,72) = 7.91, two-way ANOVA]. **(H)** NeuN CTCF index [Interaction *p* < 0.0001, *F*(3,72) = 47.92, two-way ANOVA]. Asterisks indicate statistical differences between groups. ^∗^*p* < 0.05; ^∗∗∗^*p* < 0.001; ^∗∗∗∗^*p* < 0.0001.

Immunohistochemical analysis of molecular markers expression in cells at different stages of neurogenesis (Pax6, Neurogenin 2, NeuroD1, NeuN) in the hippocampus revealed that their levels were dramatically affected in Aβ-treated rats and in the group of aging animals (**Figures 1A–D**). Noteworthy, EE did not affect the expression of the markers in the hippocampus of intact or sham-operated rats. EE demonstrated pronounced restorative effect on the impaired neurogenesis in the animals with AD model comparing with the elderly rats (**Figures 1E–H**). Among all the markers, most prominent changes were observed in the expression of NeuroD1 (reduction in all the tested animals subjected to EE).

**FIGURE 2:**
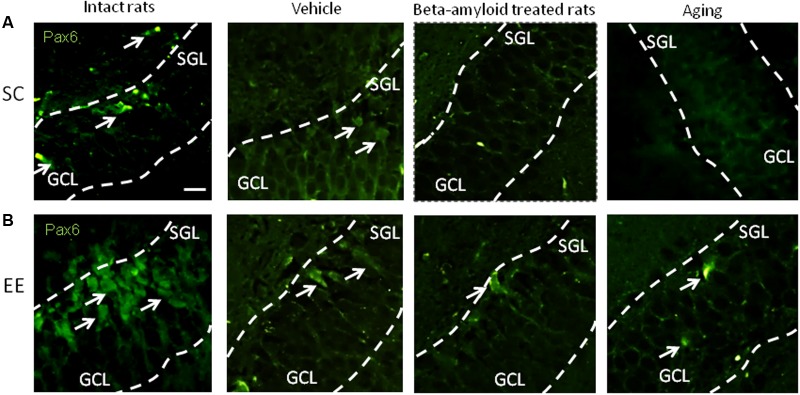
Immunohistochemical (IHC) detection of Pax6 expression in intact, vehicle-, Aβ-treated, and aged rats kept under standard conditions (SC) **(A)** and environmental enrichment (EE) **(B)**. Representative photomicrographs show Pax6 immunostaining in the granular cell layer (GCL) and subgranular layer (SGL) of dentate gyrus. Pax6 was visualized using primary anti-Pax6 monoclonal antibody and secondary chicken anti-mouse IgG conjugated with green fluorescent dye Alexa Fluor 488.

**FIGURE 3:**
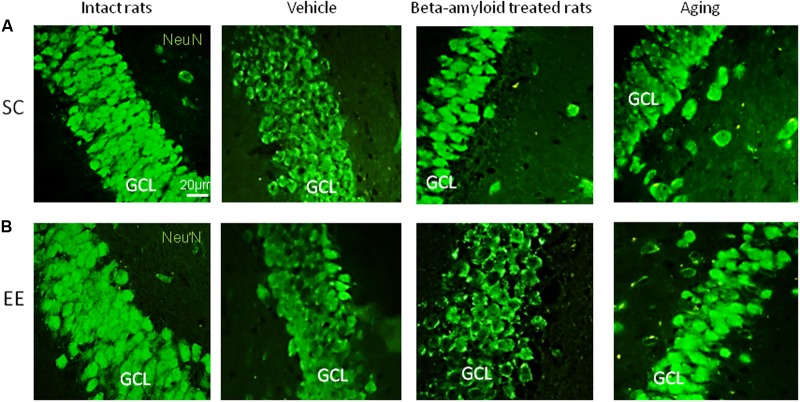
Immunohistochemical (IHC) detection of NeuN expression in intact, vehicle-, Aβ-treated, and aged rats kept under standard conditions (SC) **(A)** and environmental enrichment (EE) **(B)**. Representative photomicrographs show NeuN immunostaining in the granular cell layer (GCL) of dentate gyrus. NeuN was visualized using primary anti-NeuN antibody and secondary donkey anti-rabbit IgG conjugated with green fluorescent dye Alexa Fluor 488.

Expression of Pax6 was efficiently restored by EE in all the groups tested (**Figure 2**). It is well-known that Pax6 is a transcription factor controlling proliferation of multipotent stem/progenitor cells in hippocampus and cortex being predominantly expressed by radial glia. High expression of Pax6 is usually followed by the elevated expression of Neurogenin 2 in amplifying unipotent progenitors, which is later replaced with the expression of NeuroD1 in neuronally committed cells of hippocampus where it is required for their survival (Roybon et al., 2009). At the final stage of neurogenesis, mature neurons in the granule zone of hippocampus are highly enriched in NeuN with the evident elevation in animals kept in EE, particularly in those having AD-type of neurodegeneration (**Figure 3**).

To analyze plausible mechanisms of the observed effect of EE at the initial stage of hippocampal adult neurogenesis, we further used the experimental model suitable for the assessing the kinetic parameters of cell proliferation *in vitro*. Evaluation of NS-forming capacity of hippocampal stem/progenitor cells is rather reliable tool to get the integral pattern of neurogenic cells proliferation and differentiation in the controlled conditions where cell cluster formation and development resemble the events occurring *in vivo* (Malik et al., 2015), particularly at the earliest steps of neurogenesis that were more sensitive to the action of EE *in vivo* (as shown above). We found that being cultured *in vitro*, adult hippocampal stem/progenitor cells produce NS whose appearance could be detected and analyzed with real-time impedance measurements. Recently, we have presented actual impedance traces for NS obtained from the animals with AD model kept under SC or EE (Kuvacheva et al., 2015). For analyzing the kinetic parameters of cell proliferation we applied several assumptions shown below.

Cell index (C) which is the impedance value corresponds to the number of adherent cells, therefore, cell sedimentation leads to the elevation of cell index. If the medium viscosity is not changed, then cell sedimentation to the microplate bottom demonstrates constant velocity, and C is progressively rising at the initial phase of cell culture with the linear dependence on the time:

$$C=\eta t+C_{0}.$$

**FIGURE 4:**
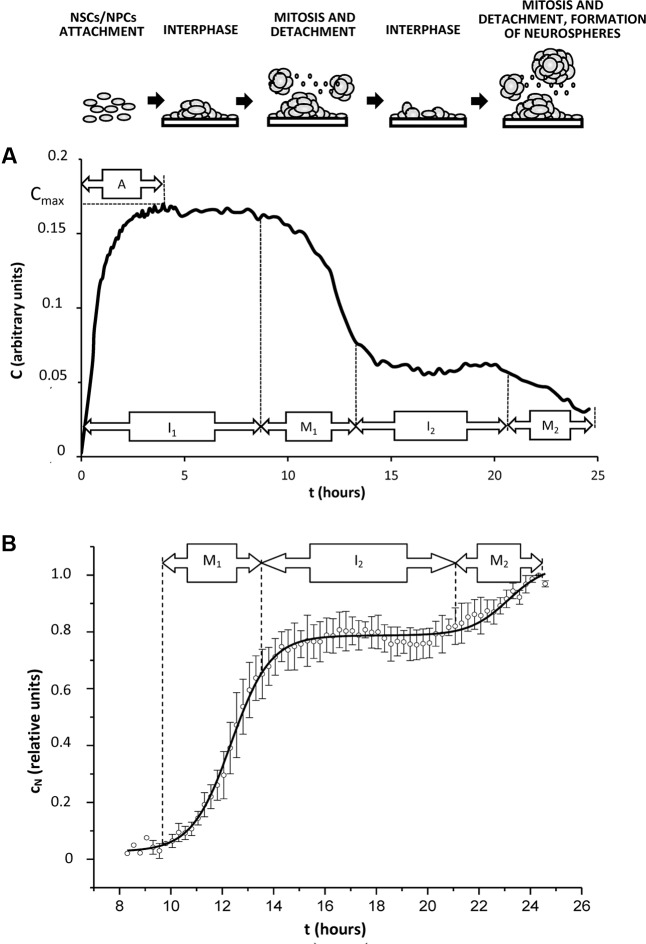
Typical kinetics of cell index in the conditions promoting generation of neurospheres *in vitro*. Cells (NSCs, NPCs) isolated from rat hippocampus were plated in the microelectrode-covered plates of xCELLigence system for impedance measurements as described in Section “Experimental Procedures.” Time of incubation is shown as t, hours. **(A)** A representative curve of cell index kinetics (indicated as C, arbitrary units) reflects real impedance trace obtained from the xCELLigence system during 24 h of cell culture. Cell index kinetics corresponds to changes in the number of attached cells during cell cycle progression. A – attachment phase, I_1,2_ – interphase of 1st or 2nd cell cycle (interphasic cells attach to the microelectrode-covered plate), M_1,2_ – mitosis in the 1st or 2nd cell cycle (mitotic cells detach from the microelectrode-covered plate). Corresponding changes in the cell culture are shown schematically at the top of the figure. **(B)** Normalized and approximated cell index (indicated as *c_n_*, relative units) was calculated from the real cell index C as shown in Section “Results,” and relates to the number of detached cells. Solid curve corresponds to the best regression model.

Constant parameter η characterizes velocity of cell sedimentation, C_0_ is the cell index obtained for the adherent cells at the initial point of measurements. The described process corresponds to the phase A (attachment of cells to the plate). Let assume that cells might be at any phase of cell cycle at this period. Phase I_1_ of the impedance trace corresponds to the period after the complete cell adhesion (interphase of the cell cycle) when maximal value of cell index (C_max_) is not changed. It is well-known that just before mitosis cell adhesion is weakened, and flattened cells acquire round shape (VanHook, 2014), therefore formation of NS leads to decrease of C value. This process corresponds to the mitotic phase M_1_. Phase I_2_ (interphase) demonstrates stable C value established just before entering mitosis in the 2nd generation of cells. This period is followed by reducing cell adhesion in the 2nd cell generation which corresponds to the mitotic phase M_2_ (**Figure 4A**).

Thus, we postulate that in the given experimental conditions: (i) cell proliferation is synchronized; (ii) mitosis-associated loss of cell adhesion leads to C decline; (iii) two generations of cells are present in the system during 24 h of cell culture on microelectrode-covered microplates.

To exclude the impact of cell sedimentation velocity on C kinetics, lets adopt the cell index value as C=C_max_ at the initial phase A. We may use the parameter shown below to take into account all the changes in C caused by the decreasing number of adherent cells:

$$C_{N}=C_{\max}-C$$

Normalization of cell index changes (c_N_) allows considering some deviations in cell concentrations in the microplate wells:

$$c_{N}=\frac{C_{N}}{C_{N\,\;\max}}$$

Typical curve for c*_N_* kinetics is shown in **Figure 4B**.

In order to analyze the observed changes in c*_N_*, we may apply a lag model developed for explaining the proliferative kinetics of cell population with the synchronized growth (Baker et al., 1998). For this purpose we must use bell-shaped distribution of cells along the different phases of cell cycle. Let’s propose that the probability of cell detachment out of microelectrode caused by alterations in adhesion mechanisms would have Boltzmann’s distribution of time, whereas normalized changes in c_N_ value could be covered by the following equation:

$$c_{N}=c_{0}+\frac{A_{1}}{1+\exp\left(\frac{t_{C}-t}{t_{M}}\right)},$$

where c_0_ – value of normalized changes of cell index at the initial point of measurements t = 0, A_1_ – amplitude of normalized changes of cell index at the point of completing the 1st cycle of proliferation, t_c_ – duration of cell cycle, t_M_ – duration of phase M_1_ (**Figure 2B**), presumably, corresponding to the duration of mitotic phase in the synchronously proliferating cell population.

The equation given above does not account for any time delay between the cell cycle initiation and beginning of cell index changes. To do this we will introduce another parameter – time-lag t_L_, and we will consider a shift from the half-time of mitotic phase duration in the previous cell division (before the measurements). Then, the equation covering cell index changes due to 1^st^ mitosis would be as follows:

$$c_{N}=c_{0}+\frac{A_{1}}{1+\exp\left(\frac{t_{C}-t_{L}-{0.5t}_{M}-t}{t_{M}}\right)}$$

Second mitosis will be accounted by introducing the second summand:

$$c_{N}=c_{0}+\frac{A_{1}}{1+\exp\left(\frac{t_{C}-t_{L}-{0.5t}_{M}-t}{t_{M}}\right)}+\frac{A_{2}}{1+\exp\left(\frac{2*t_{C}-t_{L}-{0.5t}_{M}-t}{t_{M}}\right)}$$

We have used this equation for non-linear regression analysis of experimental data. **Figure 4B** shows regression solid curve corresponding to the applied model for the cells isolated from young adult rats exposed to EE.

At the first round of regression analysis, we determined the mean value of cell cycle duration t_C_ with kinetic curves for the normalized changes of cell index for the groups of cells with the well-identified phase of 2^nd^ division. This group included data obtained from EE-treated rats as well as from aged rats housed in SC. Then, the obtained value of cell cycle duration for all the cell types was as follows:

$$t_{C}=10.8\pm 2.5hr.$$

At the second round of regression analysis, we fixed the cell cycle duration t_C_, and values of t_M_ have been estimated for all the cell types. The data obtained for the duration of “mitotic phase” t_M_ are presented in **Table 1**.

**Table 1:** Parameters of NS-forming capacity of hippocampal progenitors in rats with the Alzheimer-type neurodegeneration (AD) or physiological aging housed in the standard conditions (SC) or in the environmental enrichment (EE).

*Experimental group*	*t*_M_ *(hours)*	*Ng*	*Nc*	*K*
Young adult rats (SC)	1.23 ± 0.07	9	512	
Young adult rats (EE)	0.77 ± 0.03	14	16,384	32
AD model rats (SC)	2.23 ± 0.07	5	32	
AD model rats (EE)	1.42 ± 0.05	8	256	8
Elderly rats (SC)	1.66 ± 0.06	7	128	
Elderly rats (EE)	1.57 ± 0.08	7	128	1


*t_M_ – duration of mitotic phase M.*

*N_g_ – number of cell generations produced before full desynchronization of cell growth.*

*N_c_ – maximal cluster size obtained before full desynchronization of cell growth.*

*K – coefficient of cluster size enlargement due to effect of EE.*

Supposing t_M_ duration as a time of cell cycle “mismatch” in the synchronously proliferating population, we may assume that when the number of cell generations is $N_{g}=\frac{t_{C}}{t_{M}}$ full desynchronization would happen, thereby affecting cells microenvironment and their proliferative capacity. Under the given experimental conditions, desynchronization is most obvious in the cell culture obtained from the group of elderly rats.

Supposing doubling of cell number in every next cell generation, we may adopt that maximal cluster size (number of cells in an aggregate) which might be formed before full desynchronization of cell cycle in the culture is:

$$N_{C}=2^{N_{g}}$$

Therefore, maximal cluster size Nc should be interpreted as neurosphere-forming capacity. It is clearly shown (**Table 1**) that it is dramatically reduced in the elderly rats and – even more visibly – in the rats with AD-type of neurodegeneration. Since NS-forming ability marks the general neurogenic potential (or neurogenic reserve) of cells involved into adult hippocampal neurogenesis, we may conclude that Aβ treatment of young adult rats produces more prominent suppression of neurogenesis than healthy aging. However, the restorative potential of EE might be easily calculated using coefficient K describing changes in Nc:

$$K=\frac{N_{C}\left(EE\right)}{N_{C}\left(SC\right)}$$

As might be expected from our *in vivo* experiments, EE restored Nc more efficiently in young adult animals (fourfold greater in the control group comparing to the AD model group) but not in the elderly rats (no positive effect of EE at all). Thus, 60 days in the conditions of EE produced no any preventive effect on the aging-associated suppression of hippocampal neurogenesis *in vitro*, being, however, relatively effective in the Alzheimer’s type of neurodegeneration. This finding well-corresponds to the number of NeuN+ mature neurons in the dentate gyrus in rats kept in SC or EE (**Figure 1H**) where prominent restoration of mature neurons number was evident in the group of amyloid-treated rats but not in the group of aged animals.

## Discussion

Our findings clearly indicate that EE restores neurogenesis affected by the toxic action of Aβ or by aging at the earliest stage of niche cell development (before neuronal fate specification) as was evident both *in vivo* and *in vitro*. In contrast to aging brain, amyloid-affected brain was much more susceptible to the action of EE on the neurogenic capacity of cells. These findings confirm our hypothesis that EE may have different efficacy in AD-type of neurodegeneration and healthy aging brain in the context of neurogenesis improvement.

In accordance to previously published data (Hattiangady and Shetty, 2008) we found that expression of Pax6 within the neurogenic niche was not dramatically decreased in aging animals, but demonstrated prominent decline in AD model. However, the number of NeuN-immunopositive mature neurons was reduced both in healthy aging brain and in AD-affected brain in a similar manner. Therefore, it was not surprising that the earliest stages of neurogenesis both *in vivo* and *in vitro* were more sensitive to the action of EE. Particularly, Pax6 expression was greatly improved by EE exposure *in vivo* in Aβ-treated or aging rats, and the expression pattern of the same marker confirmed the highest neurogenic reserve in young adult rats exposed to long-lasting EE.

Unexpectedly, EE promoted obvious reduction in NeuroD1 expression in the hippocampus in AD group or aged group, whereas in the group of young adult animals no significant changes in NeuroD1 expression have been observed upon action of EE. It is known that NeuroD1 controls survival and maturation of neurons born in the adult brain (Gao et al., 2009) being under the control of Wnt-regulated activity of Sox2 and HDAC1 in NSCs (Chen and Do, 2012). This transcription factor may also direct reprogramming of glial cells into functional neurons *in vivo*, particularly, in AD brain (Guo et al., 2014). NeuroD1 overexpression could reduce functional deficits in newly-formed hippocampal neurons in the experimental model of AD (Richetin et al., 2015). In the aging brain, NeuroD expression declines in the hippocampal neurogenic niche (Uittenbogaard and Chiaramello, 2000). Thus, taking into the consideration the role of NeuroD1 in controlling cell fate and differentiation in hippocampal neurogenesis, one may assume that EE-induced reduction of NeuroD1 expression in Aβ-treated or aged rats could relate to some kind of preservation of neurogenic cells in the non-differentiated state, particularly, in the group of aging rats where elevation of NeuN+ neurons number was less evident than in the group with AD model. Since such effect on NeuroD1 expression was not found in young adult animals, changes in NeuroD1-controled cell differentiation might be considered as a “marker” of EE action in Alzheimer-type neurodegeneration or in healthy brain aging.

It should be noted that EE-mediated decline in NeuroD1 expression is in the contrast to the majority of reports on EE-induced expression of main neurogenesis markers, including NeuroD (Terada et al., 2008). However, similar effect was reported in Manuel et al. (2015) where it was attributed to the inhibition of avoidance behavior in fish subjected to EE. Thus, we may conclude that EE-induced reduction in NeuroD1 hippocampal expression in Aβ-treated or aging rats could reflect some adaptive behavioral reactions needed in further investigation.

We also should point out that housing in standard conditions revealed that NeuroD1 expression in healthy aging brain was well-preserved comparing with Aβ-affected brain. In EE-exposed rats, NeuroD1 expression underwent dramatic decline, particularly in the group of aging animals. Different sensitivity of NeuroD1-expressing cells to the action of EE in aging and AD is consistent with the observed effect of EE on the number of NeuN-immunopositive hippocampal mature neurons: stimulatory action of EE was considerably stronger in Aβ-treated rats.

In sum, EE efficiently improves neurogenesis impaired in the Alzheimer-type neurodegeneration (at least in the AD model used in our study) but produces relatively weak effect on neurogenesis in physiologically aging brain. In both cases, earliest stages of neurogenesis (stimuli-induced proliferation of stem cells and progenitors) *in vivo* represent the periods with higher sensitivity to the action of EE.

The essential question now is whether this effect might be reproduced and quantitatively explained *in vitro*? Analysis of neurogenesis *in vivo* is commonly complemented with *in vitro* studies. Along this way, establishment of NS culture *in vitro* is very useful for assessing proliferative and differentiating properties of stem cells and their progeny, however, quantitative insight into the process of NS development and evolution is highly required (Pacey et al., 2006). Characteristic behavior of NSCs/NPCs in NS culture could not be easy interpreted with simple observations and routine statistics. This problem is further complicated when a number of kinetic data appears in the conditions enabling action of various factors *in vivo* (before cell isolation) or *in vitro* (during cell clusters expansion and development). Therefore, we applied original algorithm to produce comparative analysis of EE effects on the neurogenic reserve of NSCs/NPCs obtained from all the tested groups.

We found that mathematical modeling allowed analyzing the kinetics of cell proliferation *in vitro* with high accuracy and in a good correspondence to the data obtained *in vivo*. Moreover, we were able to get the integral parameter (Nc) reflecting neurogenic reserve of stem/progenitor hippocampal cells affected by different factors (aging, amyloid treatment, EE) *in vivo*. We demonstrated that NS-forming capacity of brain stem and progenitor cells was positively affected by EE in young adult animals, aging animals, but not in elderly rats. The observed difference in the effects of EE on aging- or AD-associated impairment of neurospheres generation *in vitro* might be linked to the very recently reported age-related alterations in the duration of cell cycle (particularly, abnormal extension of G1 phase of the cell cycle not associated with a differentiation commitment) in the hippocampal stem cells resulting in neurogenesis decline (Daynac et al., 2016). A rough analogy might be found with G1-lengthening and alterations in G1/S transition in embryonic stem cells leading to imbalance between self-renewal and differentiation (Coronado et al., 2013). Therefore, the observed prevalence of desynchronization-related changes in the neurosphere-forming capacity in the group of elderly rats is very likely caused by the analogous mechanism of cell cycle impairment. Moreover, such alterations in the cell cycle progression that are evident in neurogenic cells derived from the aged brain might contribute to the relative inefficacy of EE in the context of neurogenesis recovery in aging.

In contrast to physiological aging, Aβ is able to increase NSC activity at the earliest steps of AD pathogenesis followed by later decline (Heo et al., 2007; Diaz-Moreno et al., 2013). Furthermore, EE was reported to increase Aβ accumulation in the transgenic AD model mice due to extensive synaptic activity (Jankowsky et al., 2003). Here, we will note that neurospheres-generating capacity of hippocampal stem/progenitor cells might be enhanced by neuronal excitation due to recruitment of latent stem cells into adult neurogenesis (Walker et al., 2008). That is why Aβ-induced alterations in the adult neurogenesis seem to be more profound but at the same time more sensitive to the restorative capacity of EE-provided neuronal excitation than in the healthy aging brain. Thus, it is reasonable to suggest that long-lasting exposure to EE preceding Aβ neurotoxic action results in excitation-mediated mobilization of latent stem cells and partial restoration of neurosphere-forming capacity *in vitro*. However, this effect is less prominent in Aβ-treated rats than in intact young adult animals. In the group of elderly rats, alterations in the duration of cell cycle diminish EE-induced changes in the stem/progenitor cells pool.

Previously, several attempts have been done to get a mathematical model of neurogenesis (Ashbourn et al., 2012; Ziebell et al., 2014). In this study we propose a mathematical model describing early processes in NSCs/NPCs development *in vitro*. Our time-lag model is based on several assumptions and simplifications but proves our data obtained *in vivo* and provides quantitative parameter (Nc) which is suitable for comparative analysis of effects produced by different factors (i.e., age, housing conditions, neurotoxic stimuli). Thus, combination of *in vivo* and *in vitro* approaches with mathematical modeling reveals some important characteristics of EE action on hippocampal neurogenesis in healthy aging brain or in Alzheimer-type neurodegeneration. It is tempting to speculate that the same approach might be further applied for assessing neurogenesis-targeting activity of drug-candidates in preclinical studies.

There is a growing interest to the restoration of impaired neurogenesis and prevention of neurological deficits in physiological aging (Marr et al., 2010; Mattson, 2015), but differential sensitivity of aging brain and Alzheimer’s brain to the conditions of EE should be carefully taken into the consideration. Coming back to our initial hypothesis, we may conclude that: (i) early stages of adult hippocampal neurogenesis are more sensitive to the action of EE in the experimental model of Alzheimer-type neurodegeneration or in aging; (ii) EE differently affects neurogenesis in physiological aging and in experimental AD, particularly, Aβ-affected brain is more susceptible to the action of EE comparing with healthy aging brain; (iii) different outcomes of EE action on NSCs/NPCs in AD or in healthy aging brain can be reliably detected with NS-forming assay *in vitro* and time-lag model of synchronized growth.

Thus, it is clear that EE-based neuroprotective strategies effective in the amyloid-affected brain could not be directly extrapolated to neurodegenerative processes associated with physiological brain aging. Therefore, application of EE strategy aimed to enhance adult neurogenesis should be considered as a personalized and pathogenetically based preventive or therapeutic intervention.

## Ethics Statement

All animal experiments were performed in accordance with the principles of humanity set out in the Directive of the European Community (2010/63/EU). Protocols were reviewed and approved by the Local ethics committee of the Krasnoyarsk State Medical University named after Prof. V.F. Voino-Yasenetsky (35/2011).

## Author Contributions

VS, YK, and AS conceived and designed the research. All authors performed experiments. VS, YK, NK, AM, EK, and KS analyzed data. VS, YK, OL, and EP prepared figures. VS, YK, YU, EP, and AS prepared the initial draft; VS, YK, OL, and AS revised the manuscript. All authors reviewed the final manuscript and approved its publication.

## Conflict of Interest Statement

The authors declare that the research was conducted in the absence of any commercial or financial relationships that could be construed as a potential conflict of interest.
